# Physical and oxidative stability of chicken oil‐in‐water emulsion stabilized by chicken protein hydrolysates

**DOI:** 10.1002/fsn3.1316

**Published:** 2019-12-09

**Authors:** Xianghua Chai, Kegang Wu, Chun Chen, Xuejuan Duan, Hongpeng Yu, Xiaoli Liu

**Affiliations:** ^1^ School of Chemical Engineering and Light Industry Guangdong University of Technology Guangzhou China; ^2^ School of Food Science and Engineering South China University of Technology Guangzhou China

**Keywords:** chicken protein hydrolysates, oil‐in‐water emulsions, oxidative stability, physical stability

## Abstract

The emulsifying and antioxidant properties of chicken protein hydrolysates for the physical and oxidative stabilization of chicken oil‐in‐water emulsion were investigated. The chicken protein pepsin hydrolysates obtained at reaction temperature of 33℃, 1.8% enzyme addition, liquid–solid ratio of 5:1, and reaction time of 4h, showed the DPPH radical scavenging rate of 92.12% and emulsion stability index of 0.07. The hydrolysate exerted significantly improved antioxidant activity and emulsion ability compared to the native chicken protein. The amino acid composition analysis indicated that the contents of hydrophobic amino acids including tyrosine, phenylalanine, and tryptophan were increased after hydrolysis, which contributed to the higher hydrophobicity and antioxidant activity of chicken hydrolysates. The results suggested that the chicken protein hydrolysates could be used as an alternative protein emulsifier for the production of oxidatively stable chicken oil‐in‐water emulsion.

## Graphical abstract

The chicken hydrolysates exhibited good antioxidant activity and emulsion ability, which can be used to protect chicken oil from oxidation.

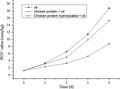

## 1 INTRODUCTION

Chicken oil presenting a unique flavor has been widely used in the seasoning products, chicken meatballs, and sausages (Anil Kumar & Viswanathan, 2013). Nevertheless, the oxidative stability of the unsaturated fatty acids restricts their use in food for the lipid oxidation negatively affects the odor, color, texture, and nutritive value (Chen et al., 2019, 2018; Yang et al., 2016). It has been proved efficient to incorporate chicken oil into liquid foods like the oil‐in‐water emulsions for that the incorporation may protect the chicken oil from oxidation through physical barrier between the oil and metal ions or oxygen (Let, Jacobsen, & Meyer, 2007; Zhang, Li, et al., 2019). This suggested that the lipid oxidation in the emulsion was determined by the chemical and physical properties of the interface. According to the reports, the properties of the interface can affect many factors like antioxidants and homogenization conditions, while the emulsifier employed affected most (McClements & Decker, 2000). This indicated that the emulsifiers with excellent antioxidant activity may inhibit the lipid oxidation by delaying the autoxidation which initiated at the interface in oil‐in‐water emulsions.

Among the emulsifiers, proteins are commonly used in the emulsion for their amphiphilic characters that offer steric or electrostatic repulsion to stabilize the oil droplets (Berton‐Carabin, Ropers, & Genot, 2014; Wang et al., 2019). For the function, bioactivity and nutrition multiple properties of milk proteins, the whey protein isolate, and sodium caseinate are normally used in the food industry (Adjonu, Doran, Torley, & Agboola, 2014). However, more and more researchers focused on the cheaper sources of proteins with good antioxidant activity and emulsion ability. Many studies have reported that the plant proteins can offer the production of physically stable emulsion like pea, soy, and lupine (Benjamin, Silcock, Beauchamp, Buettner, & Everett, 2014; Chalamaiah, Jyothirmayi, Diwan, & Dinesh Kumar, 2015; Embiriekah, Bulatović, Borić, Zarić, & Rakin, 2018; Rajabzadeh, Pourashouri, Shabanpour, & Alishahi, 2018). In addition, the protein from animal is another promising possibility applied in the emulsion, which are easily accepted by the consumer for its high nutrition value. Taherian et al. have reported that the fish protein such as gelatin and cod extracts can be developed as emulsifier (Tamm et al., 2015). Furthermore, after enzymatic hydrolysis, the fish protein can be modified to enhance their emulsifying ability and antioxidant activity (Garcia‐Moreno, Guadix, Guadix, & Jacobsen, 2016). To the best of the knowledge, there are no previous reports about the evaluation of the chicken protein which has long been commonly consumed.

Thus, this research aimed to determine the emulsifying and antioxidant properties of chicken protein hydrolysates. Furthermore, we further investigated the influence of chicken hydrolysates as emulsifiers with antioxidant effect at the interface on the physical and oxidative stability of chicken oil‐in‐water emulsions.

## 2 MATERIALS AND METHODS

### 2.1 Materials

Chicken meat was grinded by a high‐speed tissue homogenizer and kept in −18℃ freezer for further use. Chicken fat was obtained from Bewaga Foods Co., Ltd (China).

1,1‐diphenyl‐2‐picrylhydrazyl (DPPH) was purchased from Sigma‐Aldrich (USA). Maltodextrin was obtained from Lihua Starch Co., Ltd (China). Papain (900,000 U/ g) and flavourzyme (120 U/g) were obtained from Guangzhou Huaqi Biotechnology Co., Ltd (China); neutral protease (1600 AU/g), exoprotease (500,000 HUT/g), and alkali protease (580,000 DU/g) were obtained from Genencor International Ltd. (USA); composite protease (1.5 AU/g) was obtained from NOVOZYMES (USA); pepsin (1200 U/g) was obtained from Shanghai Sinopharm Chemical Reagent Co., Ltd. (China); and trypsin (4000 U/g), bromelain (800,000 U/g), and acid protease (50,000 U/g) were obtained from Auspicious New Biological Pharmaceutical Co., Ltd. (China). All other chemicals and solvents used were of analytical grade.

### 2.2 Preparation of chicken hydrolysates

A given mass of grinded chicken meat was homogenized with distilled water until reaching a final volume of 0.5 L, followed by the addition of pepsin. After completion of hydrolysis, samples were heated at 100℃ for 5 mines to deactivate the enzyme. Followed by centrifugation (5,000 g) for 10 min, the remaining solids and residual oil were removed, and the samples were stored at −80℃ for further use.

### 2.3 Single‐factor design for MFP extraction

The single‐factor design was used to determine the preliminary range of the extraction factors including X_1_ (enzyme dose: 0.5%, 1.0%, 1.5%, 2.0%, 2.5%), X_2_ (reaction temperature: 25, 30, 35, 40, 45℃), X_3_ (pH: 1.0, 1.5, 2.0, 2.5, 3.0 W), X_4_ (liquid–solid ratio: 2, 4, 6, 8, 10 min), and X_5_ (reaction on time: 1 h, 3 h, 5 h, 7 h, 9 h) (Chen, You, Abbasi, Fu, & Liu, 2015).

### 2.4 Optimization experimental design

On the basis of the single‐factor experiment, Design‐Expert software (version 8.0.5) was applied to experimental design, data analysis, and model building. A three‐level, five‐factor was applied to optimization. The whole design comprising of 18 experimental runs was carried out in a certain order as shown Table 1. All trials were performed in triplicate.

**Table 1:** Orthogonal experiment for chicken protein hydrolysis conditions

Factors		Unit	Symbols	Level of factors			
				−1		0	1
Time		h	X_1_	4		5	6
Enzyme dose		%	X_2_	1.8		2	2.2
Temperature		℃	X_3_	33		35	37
Liquid–solid ratio		‐	X_4_	1:3		1:4	1:5
pH		‐	X_5_	2.8		3	3.2
Std. order	X_1_	X_2_	X_3_	X_4_	X_5_	y_1_	y_2_
						SI	DPPH scavenging rate
1	−1	−1	−1	−1	−1	3.70	87.08
2	−1	0	0	0	0	5.87	93.93
3	−1	1	1	1	1	5.00	91.68
4	0	−1	−1	0	0	1.35	93.12
5	0	0	0	1	1	7.09	83.14
6	0	1	1	−1	−1	1.21	91.68
7	1	−1	0	−1	1	6.55	94.65
8	1	0	1	0	−1	3.41	96.61
9	1	1	−1	1	0	2.42	92.91
10	−1	−1	1	1	0	6.56	95.52
11	−1	0	−1	−1	1	8.67	93.79
12	−1	1	0	0	−1	6.77	97.00
13	0	−1	0	1	−1	6.35	94.11
14	0	0	1	−1	0	7.67	91.71
15	0	1	−1	0	1	8.80	95.45
16	1	−1	1	0	1	8.55	95.41
17	1	0	−1	1	−1	4.74	73.65
18	1	1	0	−1	0	9.79	94.48

	Sum of squared deviations	Freedom degree	Average variance	F Value	F’ value	Significance
X_1_	28.81	2	14.41	10.08	6.94	‐
X_2_	1.78	2	0.89	0.62	6.94	‐
X_3_	15.01	2	7.51	5.25	6.94	‐
X_4_	2.46	2	1.23	0.86	6.94	‐
X_5_	1.5	2	0.75	0.52	6.94	Significant

### 2.5 Antioxidant activity determination

The DPPH radical scavenging activity was measured to determine the antioxidant activity by Multi‐Mode Detection Platform (SpectraMax i3, Austria) according to Chen et al (Chen et al., 2016; Chen, Zhang, Huang, Fu, & Liu, 2017; Zhang, Chen, & Fu, 2019). Briefly, the sample was dissolved in distilled water to obtain different concentrations. Then, 200 μl of sample solution was mixed with 200 μl of ethanolic solution of DPPH. After incubation at room temperature for 30 min, the absorbance was recorded at 515 nm. Distilled water without chicken sample was used as control. DPPH radical scavenging rate was calculated by the equation:

$$\text{DPPH radical scavenging rate}\left(\%\right)=\left[1-\left(A_{2}-A_{3}\right)/A_{1}\right]\times 100$$

A_1_—the absorption of distilled water instead of sample; A_2_—the absorption of sample and DPPH in ethanol; and A_3_—the absorption of each sample and ethanol. The IC_50_ value defined as the concentration of sample to scavenge DPPH by 50% was calculated for each.

### 2.6 Analysis of emulsion stability index (SI)

As previously reported (Zhu, Qiu, Zhang, Cheng, & Yin, 2018), the oil‐in‐water emulsion was prepared by mixing the chicken protein hydrolysates with the chicken oil at the ratio of 3:1 and stirred at room temperature for 20 min, followed by a homogenization process at 9996 *g* for 2 min using a high‐speed shear machine. The stability of the emulsion was analyzed by Turbiscan Lab dispersion stability analyzer. The emulsion sample was scanned every 3 min for 1 hr at 25℃. The TSI (turbiscan stability index) is the sum of all scan differences and could be calculated according to the following equation.

$$\text{TSI}=\sqrt{\frac{\sum_{i=1}^{n}{\left(X_{i}-X_{T}\right)}^{2}}{n-1}}$$

where X_i_ is the average backscattering for each minute of measurement, X_T_ is the average X_i_, and *n* is the number of scans. The lower the TSI value, the more stable the emulsion. The TSI value was used to express the stability index (SI).

### 2.7 Analysis of the amino acid composition

The amino acid composition was determined by reference as previously reported (Cheetangdee & Benjakul, 2015). In brief, the sample (0.1 g) was hydrolyzed by HCl (6 M) at 100℃ for 24 hr. After cooling to room temperature, the excess HCl was removed by rotary evaporation. Then, derivatization of amino acids was done by phenylisothiocyanate. After the sample and standard dissolved in 100 buffer, 5 ml of the solutions was analyzed by reverse‐phase HPLC (PerkinElmer, Shelton, CT, USA).

### 2.8 Determination of surface hydrophobicity (H_0_)

Surface hydrophobicity (H_0_) was determined by the hydrophobicity fluorescence probe 1‐anilino‐8‐naphthalenesulfonate (ANS) as previously reported with minor modifications (Hayakawa & Nakai, 1985). The sample or protein was prepared at different concentrations from 0 to 0.1 mg/ml in phosphate buffer pH 7. After mixing with the ANS solution (8 mM), the fluorescence intensities were determined by a RF‐5301 PC spectro‐fluorometer (Shimadzu Corp.) with excitation wavelength at 390 nm and emission wavelength at 518 nm, respectively. The surface hydrophobicity (H_0_) was determined using a slope of linear regression between fluorescence intensity and protein concentration.

### 2.9 Oxidative stability test

The hydrolysates were added into the chicken oil with different concentrations (1%–5%), and the mixture was placed under 60 ℃ for accelerated oxidation. The peroxide value (POV) of chicken oil was measured each day for 4 days using colorimetric ferric‐thiocyanate method as previously described. The ascorbic acid was used as the control (Garcia‐Moreno et al., 2016).

### 2.10 Statistical analysis

The data were expressed as mean ± *SD* (standard deviation), and the SPSS 2.0 (Chicago, USA) was applied for one‐way analysis of variance (ANOVA). Duncan's test was used to evaluate the significance at a level of 0.05.

## 3 RESULTS AND DISCUSSION

### 3.1 Single‐factor experiments of chicken protein hydrolysates

#### 3.1.1 Effect of enzyme dose on the properties of hydrolysates

**Figure 1:**
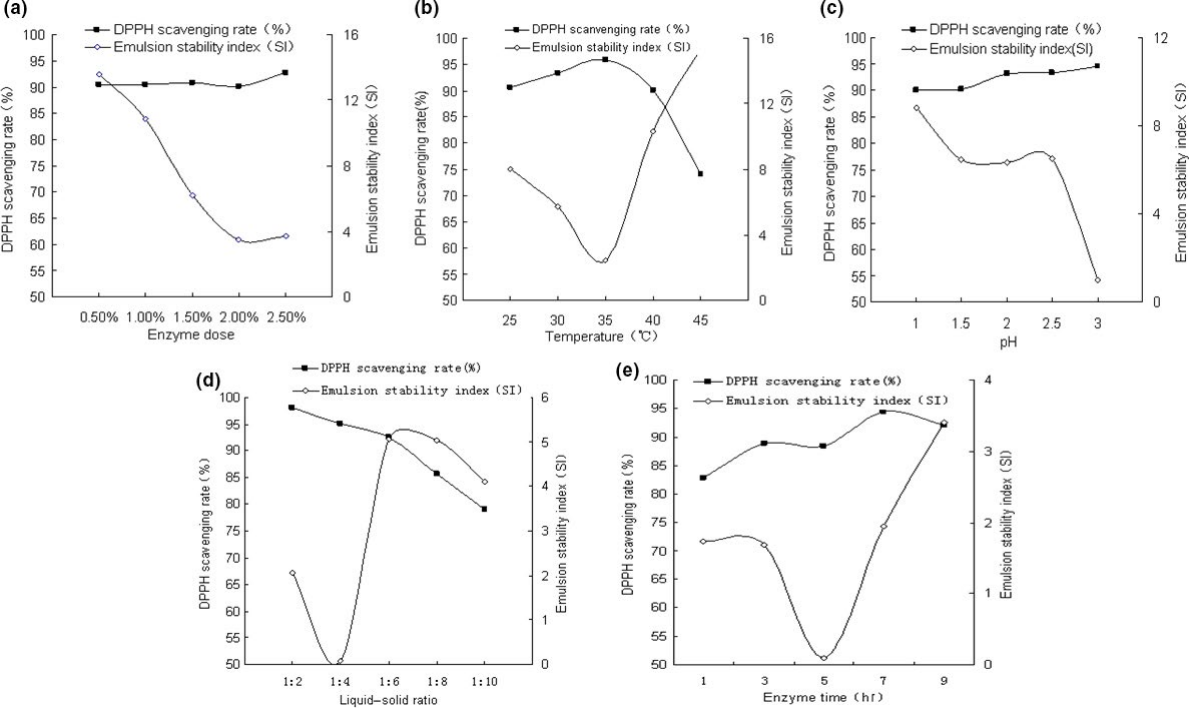
Effect of hydrolysis conditions on the properties of hydrolysates: (a) Enzyme dose; (b) Temperature; (c) pH; (d) Liquid–solid ratio; (e) Enzyme time

As shown in Figure 1a, the DPPH scavenging rate was not significantly affected by pepsin dose, while the emulsion stability index was decreased as the pepsin dose was increased. When pepsin dose was increased over 2%, emulsion stability index did not change significantly. These results indicated that pepsin dose had different impact on antioxidant activity and emulsion stability that the pepsin only acts on the aromatic amino acid‐containing peptide bonds (Paraman, Hettiarachchy, Schaefer, & Beck, 2007). Increase in pepsin dose may enhance the hydrophobicity of hydrolysates due to the elevation of the hydrophobic aromatic amino acids.

#### 3.1.2 Effect of temperature on the properties of hydrolysates

Elevation of temperature from 25℃ to 35℃, the DPPH scavenging rate increased and emulsion stability index reduced (Figure 1b). This may be due to the increased exposure of hydrophobic and antioxidant amino acids. When the temperature was increased over 40℃, pepsin activity was decreased, leading to the reduction in DPPH scavenging rate and increases in emulsion stability index. These results are consistent with the studies on pepsin hydrolysates of duck meat (Wang, Huang, Chen, Huang, & Zhou, 2015).

#### 3.1.3 Effect of pH on the properties of hydrolysates

Obviously, with the increase in initial pH from 1 to 3, DPPH scavenging rate was slightly increased, but emulsion stability index was significantly decreased (Figure 1c). During the process of hydrolysis, pH is progressively decreased. As the optimal pH for pepsin is from 1 to 3, higher initial pH is favorable to hydrolysis reaction.

#### 3.1.4 Effect of liquid–solid ratio on the properties of hydrolysates

With the increase in liquid–solid ratio, DPPH scavenging rate was decreased due to the reduction in hydrolysates content (Figure 1d). Higher liquid–solid ratio led to significant increase in emulsion stability index. However, extreme low liquid–solid ratio is also not favorable to the emulsion stability. Previous studies have also shown that extreme high or low water content is not favorable to the emulsion stability of pepsin hydrolysates (Hmidet et al., 2011).

#### 3.1.5 Effect of time on the properties of hydrolysates

As shown in Figure 1e, with the increase in the hydrolysis time, the antioxidant amino acids were progressively exposed, leading to the overall increase in DPPH scavenging rate, which is consistent with previous studies (Pownall, Udenigwe, & Aluko, 2010). In addition, with the extension of hydrolysis time, the emulsion stability of the chicken protein hydrolysates was increased, which may be due to the increase in the small peptides with terminal hydrophobic amino acids. However, extreme long reaction time may lead to overhydrolysis. Thus, the peptide molecule with emulsion property became smaller and was even hydrolyzed into amino acids, which reduces the emulsion stability of pepsin hydrolysates (Hmidet et al., 2011).

### 3.2 Optimization of chicken protein hydrolysis condition

**Figure 2:**
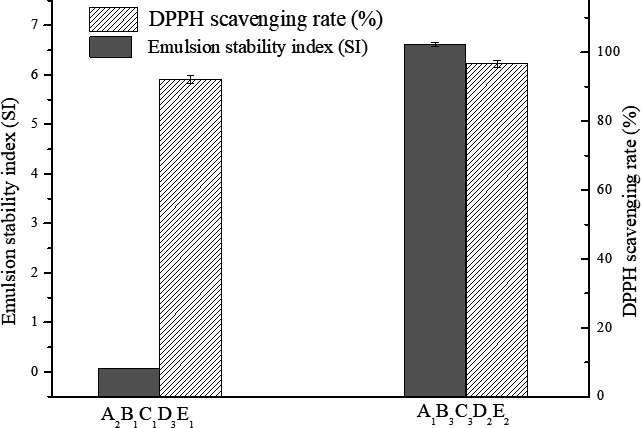
The DPPH scavenging rate and emulsion stability index of chicken protein hydrolysates obtained at different conditions

As shown in Table 1, the chicken protein hydrolysis condition was optimized by orthogonal experiment. The optimal condition for emulsion stability was A_2_B_1_C_1_D_3_E_1_, which was pH 3, pepsin dosage of 1.8%, temperature of 33℃, solid–liquid ratio of 1:5, and the reaction time of 4 hr. The order of conditions that affects emulsion stability of chicken hydrolysates was reaction time > temperature>liquid–solid ratio > enzyme dosage > pH. These results indicated that the reaction time had the most significant impact on the emulsion stability, which is consistent with previous studies (Noomen et al. 2011). The optimal condition for DPPH scavenging rate was A_1_B_3_C_3_D_2_E_2_, which is pH 2.8, pepsin dosage of 2.2%, temperature of 37℃, solid–liquid ratio of 1:4, and the reaction time of 5h. Solid–liquid and enzyme dosage affect the DPPH scavenging rate (*F* = 0.25) but not significantly. In addition, the reaction time, temperature, and pH had no significant effect on the DPPH scavenging rate. Overall, the impact of all the factors on emulsion stability was greater than that on antioxidant activity. The results shown that the optimal condition for DPPH scavenging was A_1_B_3_C_3_D_2_E_2_, and the optimal condition for emulsion stability was A_2_B_1_C_1_D_3_E_1_. However, under the optimal condition for emulsion, the chicken hydrolysates also had high antioxidant activity (Figure 2), which is consistent with the variance analysis showing that the impact of the factors on emulsion stability was higher than that on antioxidant activity. Therefore, A_2_B_1_C_1_D_3_E_1_ was selected as the optimal condition for pepsin hydrolysis of chicken protein.

### 3.3 Amino acid composition and hydrophobicity

As shown in Table 2, after the chicken protein hydrolyzed by pepsin, a significant difference in amino acid composition was observed. Obviously, the content of Leu, Tyr, Phe, Arg, and Try all increased after hydrolysis. Especially, the contents of Leu and Phe were 2.08 and 2.96 times than that of before. The results may be related to the fact that these amino acids may be the acting cites of peptide bonds when cleaved by pepsin (Chalamaiah et al., 2015). This is in accordance with the previous report that the whey protein hydrolyzed by pepsin produced more Phe, Tyr, Trp, and Leu (Embiriekah et al., 2018). Notably, the increased amino acids including Leu, Tyr, Phe, Arg, and Try were hydrophobic amino acids.

**Table 2:** Amino acid composition and content

Amino acids	Content (%)	
	Chicken protein	Chicken protein hydrolysates
Aspartic acid (Asp)	9.78	4.81
Threonine (Thr)	4.89	3.85
Serine (Ser)	4.09	5.03
Glutamic acid (Glu)	16.21	11.76
Glycine (Gly)	5.14	3.64
Alanine (Ala)	6.28	6.84
Valine (Val)	4.94	2.35
Methionine (Met)	2.94	5.13
Isoleucine (Ile)	4.79	2.14
Leucine (Leu)	8.23	17.11
Tyrosine (Tyr)	3.74	4.71
Phenylalanine (Phe)	4.34	12.83
Histidine (His)	3.14	1.60
Lysine (Lys)	9.18	2.46
Arginine (Arg)	7.03	9.52
Proline (Pro)	4.09	2.89
Tryptophan (Trp)	1.25	2.67

**Figure 3:**
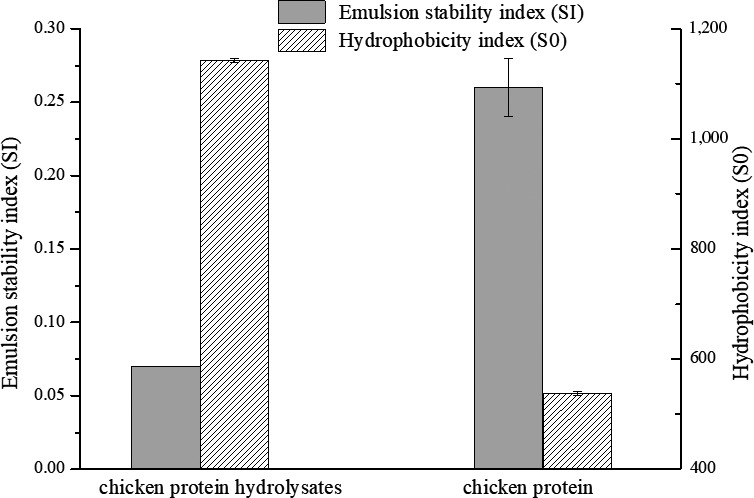
The emulsion stability index and hydrophobicity index of chicken protein hydrolysates and untreated chicken protein

Based on the results in Figure 3, the hydrophobicity index of chicken protein was 527.07, while that of chicken protein hydrolysates was increased to 1,142.5. Meanwhile, compared with the chicken protein, the emulsion stable index of hydrolysates was decreased from 0.26 to 0.07, which was consistent with the increase in hydrophobicity index. The results suggested an improvement of hydrophobicity of chicken protein after pepsin hydrolysis, which was in accordance with the increase in hydrophobic amino acids like tyrosine, phenylalanine, and tryptophan.

### 3.4 Oxidative stability of oil added with chicken protein hydrolysates

**Figure 4:**
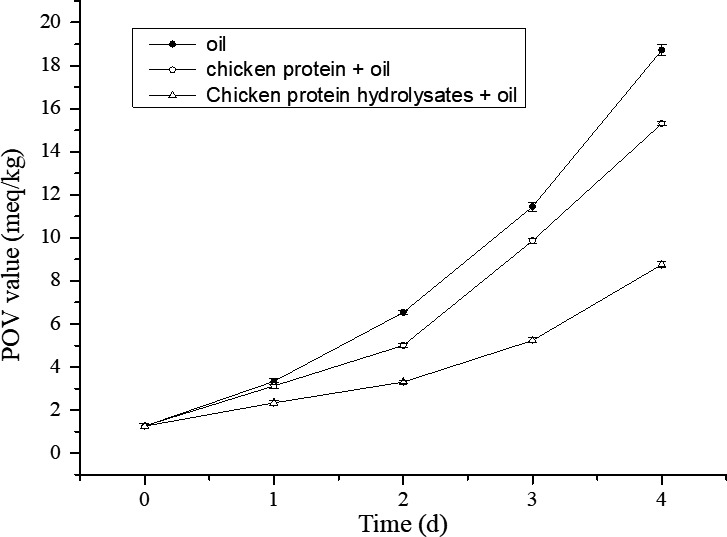
Peroxide value (POV) of the oil during storage at 60℃

The POV value of chicken oil was monitored during 4 days of storage at high temperature (Figure 4). At initial (0 day), the POV value of the chicken oil was 1.26 meq/kg. With the time increasing, the POV values of the oil showed different degree increase. For the oil without anything addition, the POV value increased rapidly to 18.72 meq/kg at the 4th day. The POV value of oil added with chicken protein increased to 15.31 meq/kg at the 4th day, which indicated that the chicken protein had a slight antioxidant activity. However, the POV value of oil added with chicken protein hydrolysates increased to 8.76 meq/kg at the 4th day, which was lower than others. The results suggested that the chicken protein hydrolysates had strong antioxidant activity, which was consistent with the good DPPH radical scavenging capacity. The improvement of the antioxidant activity of chicken protein after hydrolysis may be related to the amino acids that it has been reported that tryptophan and tyrosine, or short peptides containing histidine, tryptophan, and tyrosine had antioxidant capacity (Cheng, Xiong, & Chen, 2010a, 2010b; Hagen, Frost, & Augustin, 1989; Je, Park, & Kim, 2005).

## 4 CONCLUSION

Chicken protein hydrolysates are promising alternative emulsifiers to stabilize chicken oil‐in‐water emulsions. The chicken hydrolysates with radical scavenging rate of 92.12% and emulsion stability index of 0.07 were produced under the optimal pepsin hydrolysis condition which was enzyme dose of 1.8%, temperature of 33℃, pH of 3, liquid–solid ratio of 1:5, and reaction time of 4h. Pepsin hydrolysis leads to the exposure of antioxidant and hydrophobic amino acids, which enhances the antioxidant and emulsion activity.

## CONFLICT OF INTEREST

Authors declare they have no conflicts of interest.

## ETHICAL STATEMENT

This article does not contain any studies with human or animal subjects performed by any of the authors.
